# A Programmable Ambient Light Sensor with Dark Current Compensation and Wide Dynamic Range

**DOI:** 10.3390/s24113396

**Published:** 2024-05-24

**Authors:** Nianbo Shi, Jian Yang, Zhixiang Cao, Xiangliang Jin

**Affiliations:** 1 School of Physics and Electronics, Hunan Normal University, Changsha 410081, China; shinianbo2022@163.com (N.S.); 15226495192@163.com (J.Y.); caozhixiang1998@163.com (Z.C.); 2 Key Laboratory of Physics and Devices in Post-Moore Era, College of Hunan Province, Changsha 410081, China

**Keywords:** ambient light sensors, dark current compensation, wide dynamic range

## Abstract

Ambient light sensors are becoming increasingly popular due to their effectiveness in extending the battery life of portable electronic devices. However, conventional ambient light sensors are large in area and small in dynamic range, and they do not take into account the effects caused due to a dark current. To address the above problems, a programmable ambient light sensor with dark current compensation and a wide dynamic range is proposed in this paper. The proposed ambient light sensor exhibits a low current power consumption of only 7.7 µA in dark environments, and it operates across a wide voltage range (2–5 V) and temperature range (−40–80 °C). It senses ambient light and provides an output current proportional to the ambient light intensity, with built-in dark current compensation to effectively suppress the effects of a dark current. It provides a wide dynamic range over the entire operating temperature range with three selectable output-current gain modes. The proposed ambient light sensor was designed and fabricated using a 0.18 µm standard CMOS process, and the effective area of the chip is 663 µm × 652 µm. The effectiveness of the circuit was verified through testing, making it highly suitable for portable electronic products and fluorescent fiber-optic temperature sensors.

## 1. Introduction

With the advancements in optoelectronic information technology, microelectronics technology, and communication technology, ambient light sensors, as key devices for photoelectric detection technology, are widely used in consumer electronics, automotive electronics, and industrial control. More and more electronic products have entered and changed our daily lives, such as multimedia music players, smartphones, digital cameras, tablet PCs, and car navigation systems [1,2,3,4,5], all of which use ambient light sensors to convert light illumination into electrical signals to prolong battery life and backlight life, as well as improve power efficiency. These portable electronic devices have become an indispensable part of our lives and work, making our lives more convenient and our quality of life higher. Photoelectric sensors are ubiquitous in portable electronics, and they have transitioned successfully from industrial, medical, and aerospace applications to the consumer electronics market [6,7].

Ambient light sensors detect light signals based on the photoelectric effect, converting them into corresponding electrical signals. An ambient light sensor comprises two main parts: a photodetector and a conversion circuit. The photodetector generates current or voltage signals according to the ambient light intensity, and the conversion circuit processes these signals. Silicon photodiodes are widely utilized in photodetectors because of their fast response time, high sensitivity, wide range of response wavelengths, and ability to be integrated with a circuit. Although ambient light sensors have different application areas, there are some design requirements that are the same, all of which require a good spectral response, a wide ambient light range, a long lifetime, high integration, low power consumption, and a fast dynamic response. However, a serious problem for photodiodes is the sensitivity of a dark current to temperature, which directly affects the performance of ambient light sensors. For example, when the temperature rises, the dark current grows exponentially, causing the ambient light sensor to fail to function. Therefore, it is important to minimize the dark current in ambient light sensors. The literature [8] proposed an on-chip optical sensor capable of detecting the direction of incident light. The sensor was realized using a 0.5 µm standard CMOS process. The literature [9] also proposed a novel, digital ambient light sensor system that automatically adjusts the sensitivity according to the lighting conditions. The literature [10] proposed a CMOS monolithic digital light sensor with a calibration technique suitable for ambient light sensors. Additionally, the literature [11] proposed a low-power ambient light sensor with digital output, using a correlated photocurrent reflector to improve its power consumption and a pulse-width comparison algorithm to achieve an adjustable dynamic range. However, none of the works in the literature [8,9,10,11] have considered the effect due to a dark current. The literature [12] proposed an ultra-low-power ambient light sensor for portable devices fabricated using 0.5 µm standard CMOS technology. However, the dynamic range is only 10 to 1000 Lux, which limits the usage environment. The literature [13] proposed a CMOS smart light-sensing chip for automatic brightness adjustment applications. However, the dynamic range is only 30 to 300 Lux. Also, the literature [14] proposed an ambient light sensor with dark current compensation and adaptive resolution adjustment technology. However, the dynamic range is only 0 to 16 K lux. The literature [15] proposed a new, integrated ambient light sensor that can be used to detect the illumination of ambient light. However, the dynamic range is only 0 to 10 K lux.

To overcome the above problems, a programmable ambient light sensor with dark current compensation and a wide dynamic range is proposed in this paper. The proposed dark current compensation circuit effectively suppresses the effect of a dark current and provides a wide dynamic range from 0 lux to 100 K lux over the operating temperature range. The performance and effectiveness of the circuit were verified through experimental tests, and they are attractive for portable electronic devices and fluorescent fiber-optic temperature sensor applications.

## 2. The Structure and Principle of the Photodiode Device

**Figure 1:**
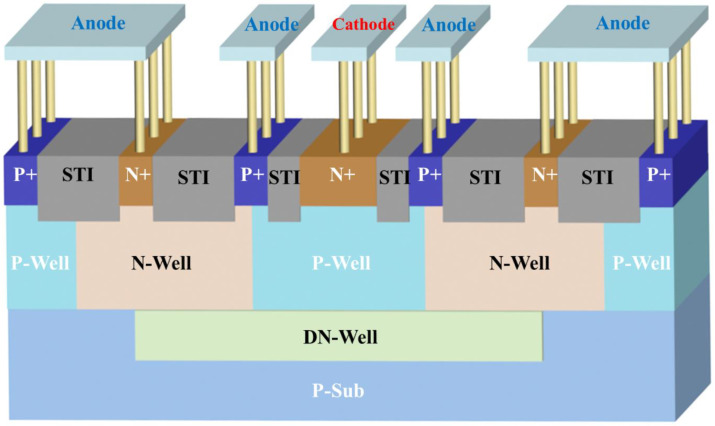
Three-dimensional structure of PD device.

The 3D structure of the photodiode (PD) device, designed and fabricated using a 0.18 µm standard CMOS process, is shown in Figure 1. The N+ region serves as the cathode of the device, and the P+ region serves as the anode of the device. From Figure 1, it can be seen that there are three vertically oriented PN junctions in the figure, one formed through the N+/P-Well contact surface, one formed via the DN-Well/P-Well, and one formed via the DN-Well/P-SUB and the inverted PN junctions formed in the edge region, which effectively mitigates the effect of the substrate noise carriers on the device and reduces the dark current. When light is irradiated on the surface of the device, photons larger than the energy of the silicon band gap will be absorbed via the material. As a result, electrons in the valence band jump to the conduction band, forming photogenerated carriers. These photogenerated carriers form a stable photocurrent under the action of electric field force.

**Figure 2:**
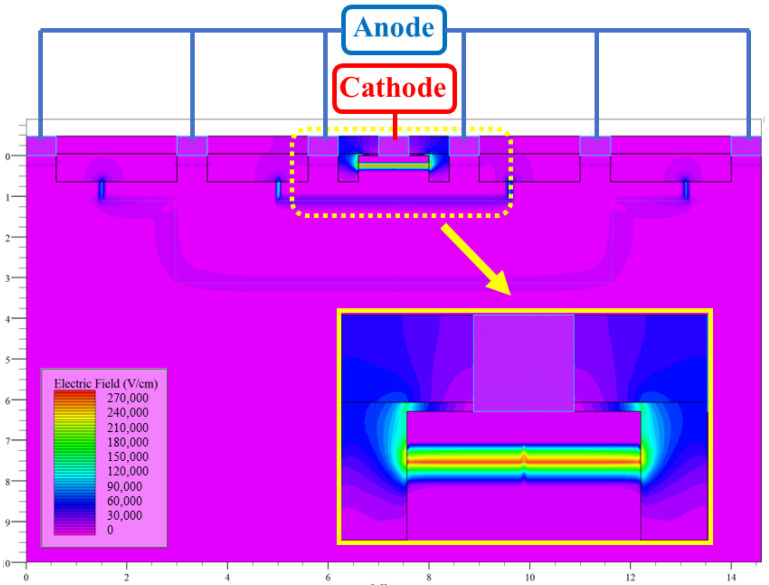
Electric field distribution of PD device.

The operating principle and structure of the device, including the electric field distribution and current distribution of the device, were simulated and verified using the silvaco TCAD tool. In the simulation, a bias voltage of 2 V was applied to the cathode of the PD device, and the anode was 0 V. Figure 2 shows the simulation results of the electric field strength during normal operation, and it can be seen that the maximum value of the electric field strength was mainly concentrated at the PN junction formed via the N+/P-Well with an electric field strength of about 2.7 × 10^5^ V/cm.

**Figure 3:**
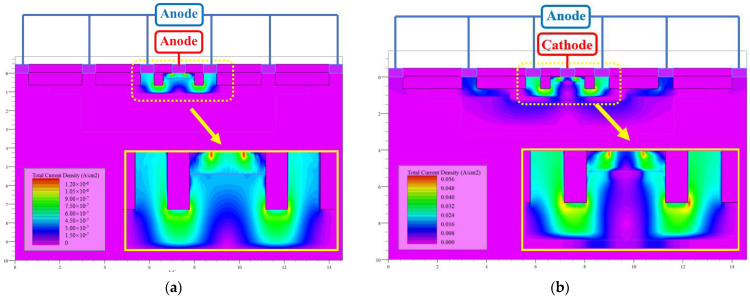
(**a**) Current distribution of PD device under dark conditions; (**b**) current distribution of PD device under light conditions.

**Figure 4:**
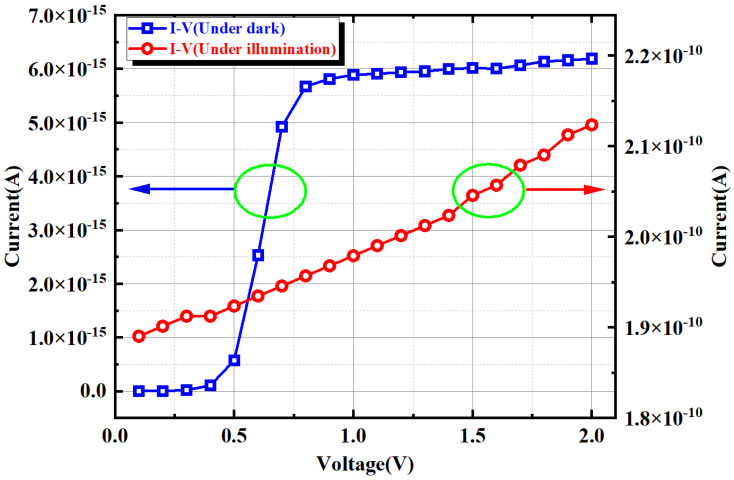
Simulated I-V characteristics of the PD device.

The total current density distributions of the PD device under dark and light conditions are shown in Figure 3. Under the light condition, the total light current density of the PD device was 5.6 × 10^−2^ A/cm^2^, which was significantly higher than the dark current density of 1.2 × 10^−6^ A/cm^2^. Figure 4 depicts the simulated current–voltage (I-V) characteristics of the PD device. The reverse-bias voltage varied from 0 V to 2 V, and the resulting I-V curves are plotted in blue for the dark condition and in red for the illuminated condition.

## 3. Design of a Programmable Ambient Light Sensor

**Figure 5:**
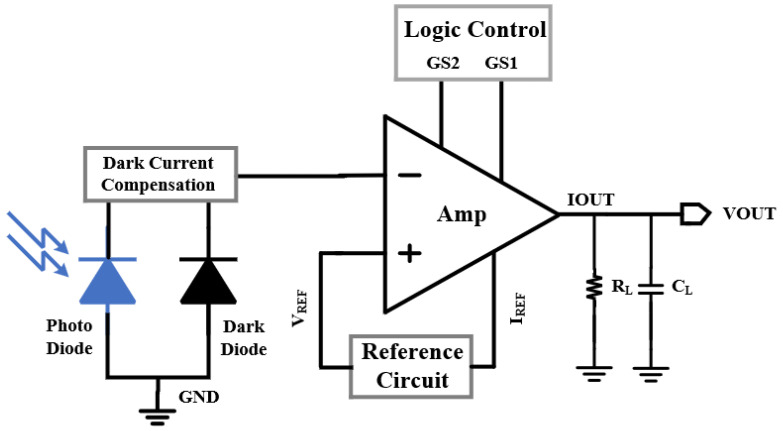
Simplified block diagram of ambient light sensor.

The main function of the ambient light sensor is to convert weak light signals into electrical signals and amplify them to the desired level in order to achieve proper signal processing in the ambient light sensor system. A simplified block diagram of the designed ambient light sensor circuit is shown in Figure 5, which mainly consists of PD_Light_, PD_Dark_, an operational amplifier, a reference-source circuit, and a logic-control circuit. The operational amplifier amplifies the output photocurrent signal of PD_Light_. The reference-source circuit provides reverse-bias voltage, V_REF_, to the PD_Light_ and the reference current to the operational amplifier. The logic-control circuit provides three selectable output-current gain modes. The PD_Light_ operates at the reverse-bias voltage, V_REF_, and shows a linear relationship between its output current and light intensity. The PD_Dark_ operates in dark conditions to compensate for the dark current. The output port I_OUT_ is connected to the resistor R_L_ to convert the current signal into the voltage signal.

In this programmable ambient light sensor, the reference-source circuit provides the reference voltage, V_REF_, and the reference current, I_REF_, for the operational amplifier. The reference current, I_REF_, enables the operational amplifier to operate properly. The reference voltage, V_REF_, is directly connected to the in-phase input of the operational amplifier. According to the characteristics of the operational amplifier, the cathode voltage of the PD_Light_ is approximately equal to V_REF_. Because the anode of the PD_Light_ is grounded, it operates under reverse-bias conditions, resulting in a current signal.

**Figure 6:**
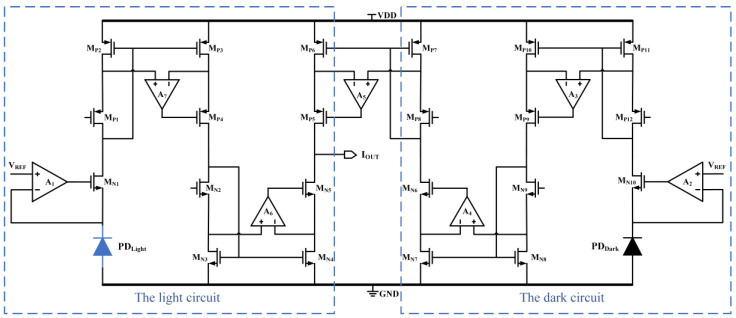
Schematic diagram of the dark current compensation circuit.

One of the characteristics of the photodiode is that, when operating under reverse-bias conditions without light, a dark current will flow through the photodiode, and this dark current needs to be compensated using a second identical photodiode. The schematic diagram of the dark current compensation circuit is shown in Figure 6, in which the PD_Light_ detects the ambient light, while the metal-covered PD_Dark_ generates the dark current for compensation. The anodes of both photodiodes are grounded, and the inverting inputs of operational amplifiers A_1_ and A_2_ are connected to the cathodes of the PD_Light_ and PD_Dark_, respectively, so that the photodiodes operate under reverse-bias conditions. The operational amplifier A_7_ in-phase input and inverse input to the M_P2_ and M_P3_ drain, respectively, when the operational amplifier A_7_ gain is very large and the M_P2_ and M_P3_ drain voltage is approximately equal, can make M_P1_-M_P4_ constitute a low-voltage, high-precision cascode current mirror accurately mirror the current. The photocurrent and dark current generated via the PD_Light_ are output to the M_N5_ drain via two modified, low-voltage, high-precision cascode current mirrors, while the dark current generated via the PD_Dark_ is output to the M_P5_ drain via three modified, low-voltage, high-precision cascode mirrors. Simply by adjusting the magnification, the two dark currents are subtracted, leaving only the light current I_OUT_ to flow into the next level of low-voltage, high-precision cascode current mirrors for amplification and output.

In this programmable ambient light sensor, the logic-control circuit provides three selectable output-current gain modes with a step size of 10. The programmable gain settings are shown in Table 1. When GS2 is 0 and GS1 is 1, the circuit operates in High Gain mode with a magnification of 100,000. When GS2 is 1 and GS1 is 0, the circuit operates in Medium Gain mode with a magnification of 10,000. When GS2 and GS1 are both 1, the circuit operates in Low Gain mode with a magnification of 1000. When GS2 and GS1 are both 0, the circuit is in Off mode. 

## 4. Experimental Results and Discussion

**Figure 7:**
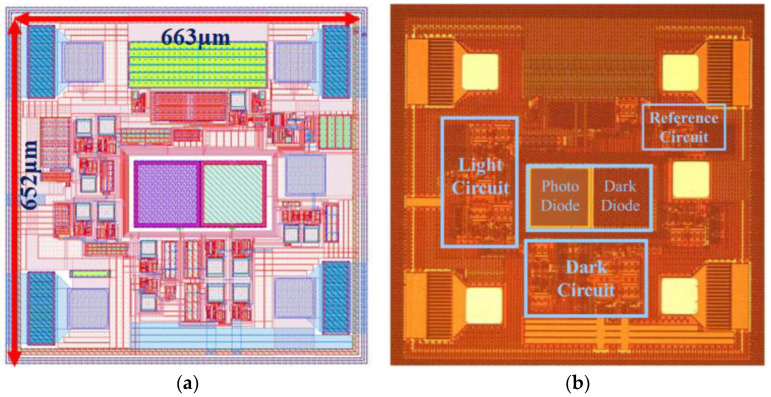
(**a**) Circuit layout of the proposed programmable ambient light sensor with dark current compensation and a wide dynamic range. (**b**) Micrograph of the circuit.

The programmable ambient light sensor with dark current compensation and a wide dynamic range proposed in this paper was designed and fabricated using a 0.18 µm standard CMOS process. The layout and micrographs of the circuit are shown in Figure 7. The effective area of the chip is 663 µm × 652 µm. 

**Figure 8:**
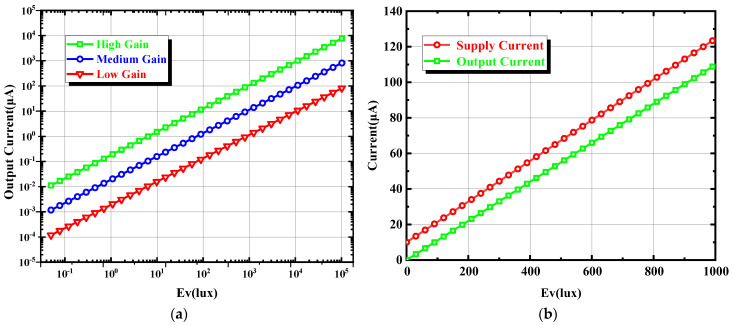
(**a**) Measurement results of output current changing with light intensity (**b**) Measurement results of power supply current and output current changing with light intensity.

The measurement results of the output current with the change in light intensity are shown in Figure 8a, which shows that, when the supply voltage is 3.3 V and the light intensity is increased from 0 lux to 100 K lux, the output current changes linearly with the light intensity, the output current increases linearly, and the step size is 10 times in three different operating modes. When the light intensity increases from 0 lux to 1 K lux, the measurement results of the power-supply current and output current with the change in light intensity are shown in Figure 8b, which shows that both the power-supply current and output current change linearly with the light intensity. From the measurement results, it can be seen that the programmable ambient light sensor proposed in this paper has a wide dynamic range and good linearity. 

**Figure 9:**
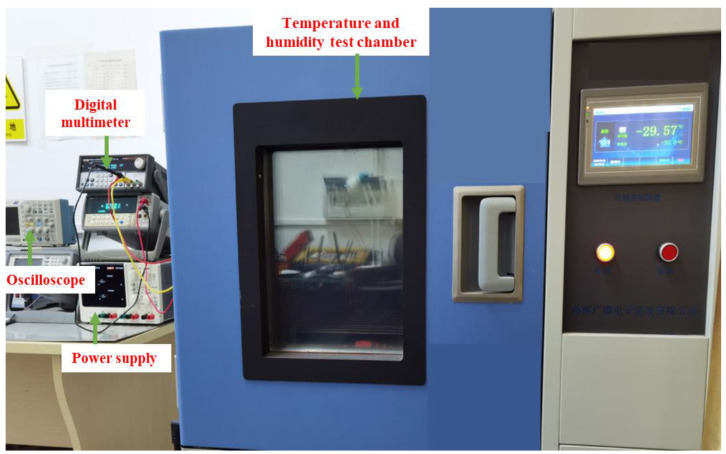
Temperature and humidity test chamber.

**Figure 10:**
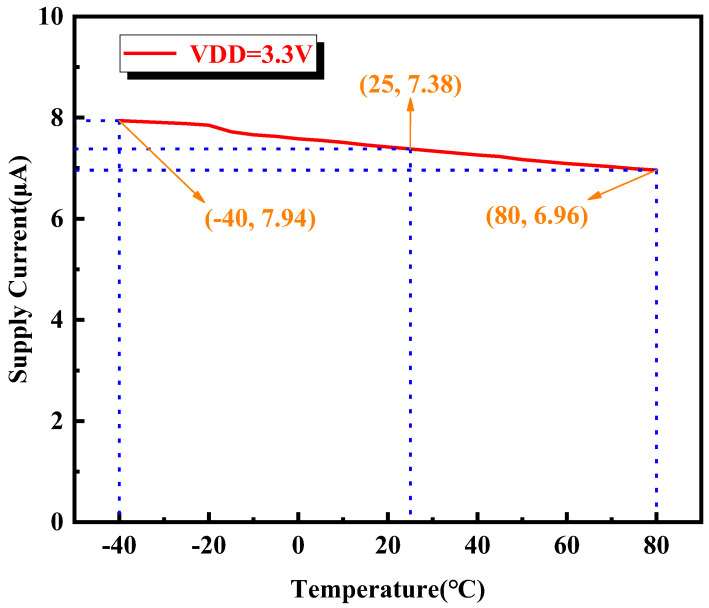
Measurement results of power supply current changing with temperature in dark environment.

The temperature characteristics of the ambient light sensor were measured using a temperature and humidity test chamber, as shown in Figure 9. The measurement results of the power supply current with temperature changes in a dark environment are shown in Figure 10. From the figure, it can be seen that, when the supply voltage is 3.3 V and the temperature is −40 °C, the maximum power supply current is 7.94 µA. When the temperature is 80 °C, the minimum power supply current is 6.96 µA. When at room temperature, the power supply current is only 7.38 µA. It can be seen from the measurement results that the programmable ambient light sensor proposed in this paper has low current consumption across the whole operating temperature range.

**Figure 11:**
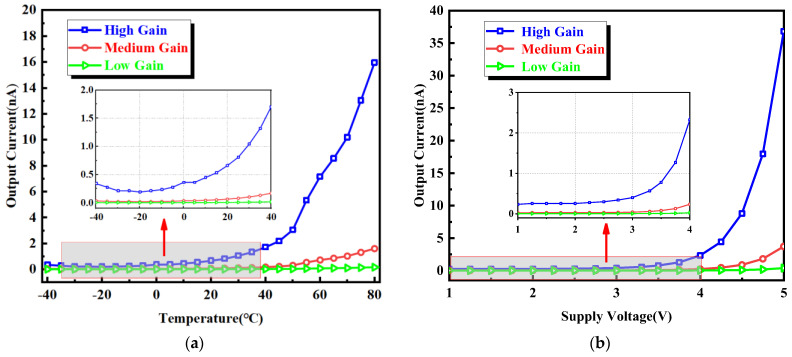
(**a**) Measurement results of output current as temperature changes. (**b**) Measurement results of output current as power-supply voltage changes.

The measurement results of the output current changing with the temperature in a dark environment are shown in Figure 11a. As can be seen from the figure, when the temperature is −40–40 °C, the output dark current is very small, and when the temperature rises to 40 °C, the output dark current begins to increase gradually. This is mainly because, as the temperature increases, the dark current of the two photodiodes increases sharply, resulting in a large difference in the size of the dark current. When the circuit works in High Gain mode, the current magnification is 100,000, and the output dark current is only 16 nA at the temperature of 80 °C. The measurement results of the output current changing with the power supply voltage in a dark environment are shown in Figure 11b. It can be seen from the figure that, when the power supply voltage is 1–4 V, the output dark current is very small, with a maximum of only 2.3 nA. When the supply voltage is increased to 4 V, the output dark current begins to rise. This is mainly because the power supply voltage is 3.3 V when the CMOS tube is working normally, and continuing to increase the power supply voltage may lead to an increase in the leakage current. The measurement results demonstrate that the proposed circuit effectively mitigates the influence of the dark current. A comparison table with previously published designs is shown in Table 2. In contrast to the present approach, none of the previously published designs proposed dark current compensation.

**Figure 12:**
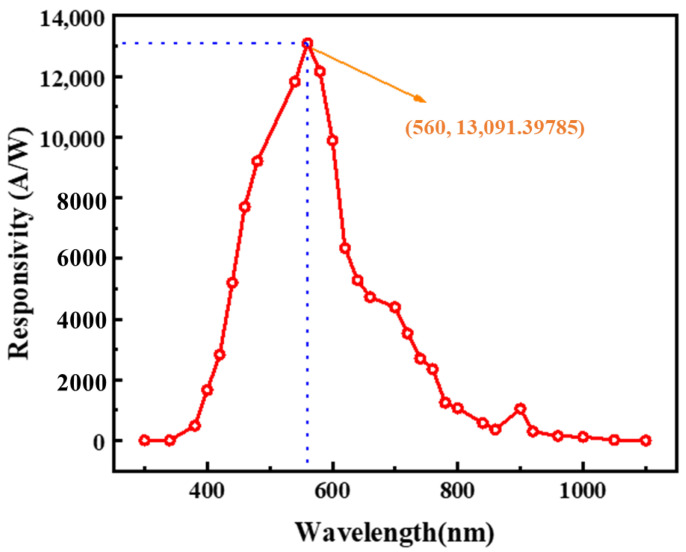
The spectral response measurements of circuits.

The output current of the circuit is recorded as the light source wavelength varies from 300 nm to 1100 nm, with a power supply voltage of 3.3 V and a PD_Light_ reverse-bias voltage of 0.8 V. The output current is then divided by the optical power at each wavelength to obtain the spectral response of the circuit, as shown in Figure 12. The optimal spectral response occurs between 500 nm and 600 nm, reaching a peak of 13,091 A/W at a wavelength of 560 nm.

## 5. Conclusions

In this paper, a programmable ambient light sensor with dark current compensation and a wide dynamic range has been proposed. The proposed ambient light sensor has a wide dynamic range from 0 lux to 100 K lux and good linearity over the operating temperature range, it has a built-in dark current compensation circuit to effectively suppress the effects caused due to a dark current, and it can operate over a wide voltage range and a wide operating temperature range. In future research, the proposed ambient light sensor will be attractive in portable electronic applications such as smartphones, cameras, and LCD televisions and monitors.

## Figures and Tables

**Table 1:** Programmable gain settings.

GS2	GS1	Mode	Magnification
0	0	Power Down	-
0	1	High Gain	100,000
1	0	Medium Gain	10,000
1	1	Low Gain	1000

**Table 2:** Comparisons with previously published designs.

	[9]	[10]	[11]	[12]	[13]	[14]	This Work
Technology	LTPS CMOS technology	0.35 µm CMOS process	0.18 µm CMOS process	0.5 µm CMOS process	0.35 µm CMOS process	0.35 µm CMOS process	0.18 µm CMOS process
Power supply	3 V	3.3 V	1 V	5 V	3.3 V	/	3.3 V
Chip area	0.011 mm^2^	1.28 mm^2^	/	1.32 mm^2^	0.735 mm^2^	0.269 mm^2^	0.43 mm^2^
Dark current compensation	None	None	None	None	None	None	None
Dynamic range	0–90 Klux	0–37 Klux	10–10,000 lux	10–1000 lux	30–300 lux	0–16 Klux	0–100 Klux

## Data Availability

The study’s data are contained within the article.
